# Advantages of Fresnel biprism-based digital holographic microscopy in quantitative phase imaging

**DOI:** 10.1117/1.JBO.25.8.086501

**Published:** 2020-08-04

**Authors:** Charity Hayes-Rounds, Brian Bogue-Jimenez, Jorge Garcia-Sucerquia, Omar Skalli, Ana Doblas

**Affiliations:** a The University of Memphis, Department of Electrical and Computer Engineering, Memphis, Tennessee 38152, USA; b Universidad Nacional de Colombia Sede Medellin, School of Physics, Medellin, Colombia; c The University of Memphis, Department of Biological Sciences, Memphis, Tennessee 38152, USA

**Keywords:** Fresnel biprism, digital holographic microscopy, quantitative phase imaging, polarization microscopy

## Abstract

**Significance:** The hallmarks of digital holographic microscopy (DHM) compared with other quantitative phase imaging (QPI) methods are high speed, accuracy, spatial resolution, temporal stability, and polarization-sensitivity (PS) capability. The above features make DHM suitable for real-time quantitative PS phase imaging in a broad number of biological applications aimed at understanding cell growth and dynamic changes occurring during physiological processes and/or in response to pharmaceutical agents.

**Aim:** The insertion of a Fresnel biprism (FB) in the image space of a light microscope potentially turns any commercial system into a DHM system enabling QPI with the five desired features in QPI simultaneously: high temporal sensitivity, high speed, high accuracy, high spatial resolution, and PS. To the best of our knowledge, this is the first FB-based DHM system providing these five features all together.

**Approach:** The performance of the proposed system was calibrated with a benchmark phase object. The PS capability has been verified by imaging human U87 glioblastoma cells.

**Results:** The proposed FB-based DHM system provides accurate phase images with high spatial resolution. The temporal stability of our system is in the order of a few nanometers, enabling live-cell studies. Finally, the distinctive behavior of the cells at different polarization angles (e.g., PS capability) can be observed with our system.

**Conclusions:** We have presented a method to turn any commercial light microscope with monochromatic illumination into a PS QPI system. The proposed system provides accurate quantitative PS phase images in a new, simple, compact, and cost-effective format, thanks to the low cost (a few hundred dollars) involved in implementing this simple architecture, enabling the use of this QPI technique accessible to most laboratories with standard light microscopes.

## 1 Introduction

Digital holographic microscopy (DHM)^1^^–^^5^ is one of the most promising quantitative phase imaging (QPI) techniques^6^ to perform live-cell imaging while providing topographical information on the specimen. The hallmarks of DHM compared with other QPI methods are high speed, stability, accuracy, spatial resolution, and polarization sensitivity (PS). Temporal-phase stability is identified as one of the most important figures of merit in QPI.^6^ Among all DHM configurations, common-path DHM systems^7^^–^^16^ provide the highest temporal stability, allowing subnanometer path-length temporal sensitivity to study dynamic events in live biological specimens. High-speed image acquisition is also critical in capturing rapid dynamic events and investigating how they are affected by external perturbations. Off-axis DHM systems are currently the fastest DHM systems since only a single recorded image is required to provide a quantitative phase image.^17^ Although all off-axis DHM systems provide rapid imaging, not all of them provide high-resolution and accurate phase images since the majority of off-axis DHM systems operate in nontelecentric mode.^18^^,^^19^ In 2014, Sánchez-Ortiga et al.^20^ demonstrated how off-axis DHM systems can operate at the diffraction limit, thereby enabling the reconstruction of diffraction-limited DHM images. On the other hand, because several quantitative biological parameters^6^ such as the integral intracellular refractive index can be estimated from a phase measurement, a key feature of any QPI system is that the response should be accurate and shift invariant. For this reason, telecentric-based DHM systems^18^^,^^19^ are the most suitable QPI systems.

Ultimately, PS imaging systems are advantageous in biological imaging increasing the understanding of cell biological processes^21^^–^^24^ since they determine the isotropic properties of different cellular and extracellular molecular components, such as microtubules and amyloid.^21^

The aim of this work is twofold. First, we demonstrate the advantages of QPI imaging using a Fresnel biprism (FB)-based DHM system. The proposed DHM system offers the desired features in QPI simultaneously: high temporal sensitivity, speed, accuracy, spatial resolution, and sensitivity to the birefringence. To the best of our knowledge, this is the first FB-based DHM system providing these five features all together. Note that the first FB-based DHM system^25^ followed a nontelecentric configuration, and, as a result, two of the above features (high accuracy and high spatial resolution) may not have been met. More recently, a FB-based DHM system has been proposed for a multiwavelength approach.^26^ In a close related work, Ebrahimi et al.^27^ proposed an off-axis lensless DHM system using a FB. Because the system follows an off-axis configuration, meaning that there is no overlay between the different orders composing the Fourier spectrum of the recorded hologram, one can retrieve both amplitude and phase distributions by means of just spatial filtering the object frequencies from the spectrum of the single hologram. In their proposed set up, the FB is illuminated by a plane wave, and therefore, generates two tilted plane waves that are focused into two-point sources using a converging lens. The focal lens of this converging lens and the biprism features (e.g., refractive index and refringence angle) dictate the separation between these two-point sources, which affects the separation of the orders in the hologram’s spectrum (e.g., the interference fringes’ period), the interference field of view (FOV), and the sample overlapping in the camera plane. A major drawback in common-path DHM systems is their applicability to investigate quantitative phase images. The differences of that work^27^ and our proposed FB-based system are the experimental resolution limit (3.48  μm^27^ versus 0.775  μm) and the accuracy of the phase images using a single-shot approach. In Ref. 27, the sample is illuminated by two slightly tilted diverging spherical waves. Although both diverging spherical waves that illuminate the sample should have the same curvature, their origins (e.g., center of the spherical wave) are different, and therefore, they would never cancel each other. In fact, note that the reconstructed phase image of the *Candida albicans* cells reported in Ref. 27 was generated after compensating the undesired spherical wavefront by *a posteriori* point-wise subtraction of the measured phase with no sample information placed from the measurement with the sample in place. The main drawback of this latter method is that it requires two shots, which may not always be possible in live cell imaging. Here, we propose a simple and effective way to provide accurate phase imaging. Regarding the polarization feature, the primary advantage of our PS-DHM system with respect to others^28^ is its compactness. Second, thanks to all the presented advantages, we want to convince the community that commercial systems could be easily integrated with a FB-based QPI-DHM system. As a result of the advantages and the ease of adapting our proposed DHM system to standard light microscopes, we predict FB-based DHM systems working together with the commercially available phase contrast techniques [e.g., phase contrast microscopy,^29^^,^^30^ differential interference contrast (DIC) microscopy,^31^ and Hoffman modulation contrast (HMC) microscopy^32^] will be routinely implemented in standard light microscopes, increasing DHM applications for a larger number of biological and biomedical studies. It is important to mention that Wollaston-prism-based DHM systems^9^ and simplified DHM systems using a Michelson interferometer approach^16^ could provide QPI images with high temporal sensitivity, speed, accuracy, and spatial resolution. Nonetheless, the applications of those systems for birefringence measurements are not direct. Other low-cost and compact DHM systems have been implemented using a Lloyd’s mirror^33^ and a lateral shearing plate.^34^ Again, the main difference of those systems with our proposed method is related to PS measurements. In particular, for the Lloyd-based DHM system,^33^ and assuming an illuminating beam whose polarization state is linear or circular, the polarization state of the reference beam, which is generated as the reflected beam from the Lloyd’s mirror, becomes elliptical. Because coherent interference only occurs when the polarization state of both beams (object and reference beams) are the same, no interference fringes are formed, resulting in no retrieval of the complex object information. For the DHM system implemented with a lateral shearing plate,^34^ both waves are reflected and have the same polarization state (e.g., elliptical polarization state) and an interference pattern is observed. However, the main inconvenience in such DHM systems^34^ is the measurement of the retardance (e.g., birefringence) map since the initial linear polarization state has been converted to an elliptical polarization state. Thereby, the recorded image will have both ordinary and extraordinary contributions with different amplitude ratios. For the estimation of the retardance map, one should decouple each component. This decoupling can be performed by inserting new elements, which compromises the original compact and low-cost advantages. For example, the measurement of the retardance map could be achieved by inserting a polarized beam splitter after the lateral shearing plate. The polarized beam splitter separates the ordinary and extraordinary components, and one could record them simultaneously using two CCD/CMOS sensors. However, using this approach, there is still a need for addressing the amplitude difference between each component due to the elliptical polarization state. Without addressing this difference, the birefringence measurements may not be accurate, resulting in misclassification and misdiagnoses of diseases using PS lateral shearing-based DHM system.

## 2 Polarization-Sensitive Digital Holographic Microscopy using a Fresnel Biprism

### 2.1 Optical Configuration

**Fig. 1.**
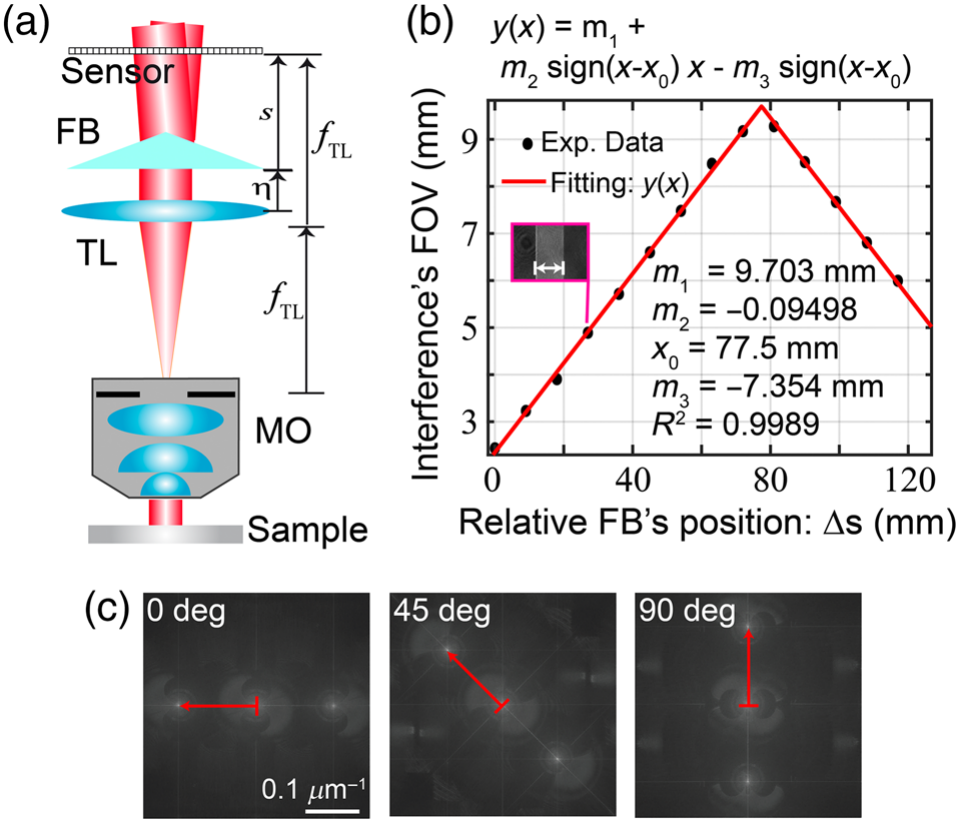
Proposed common-path digital holographic microscopy based on an FB. (a) Schematic of the system, (b) experimental interference FOV, and (c) optimization of the space bandwidth of the camera by rotating the biprism.

Figure 1 illustrates the optical configuration of the proposed common-path DHM system using a biprism. The sample is mounted onto a translation stage for focusing and is illuminated by a low-power collimated laser diode module (CPS532, Thorlabs) of wavelength 532 nm and spectral bandwidth <1  nm. The light scattered by the sample is imaged by a system composed of an infinity-corrected 40×/0.65  NA microscope objective (MO) and an achromatic tube lens (TL). The lateral magnification of the system is M=−44.44× since the focal lengths of TL and MO are 200 and 4.5 mm, respectively. It is important to mention that the proposed system operates in the telecentric regime since the aperture stop of the MO is located at the front focal plane of the TL (e.g., object focal plane). A Basler acA5472-17-μm CMOS sensor (5472×3648  pixels, ${2.4\text{-}\mu m}^{2}$ pixel size) is placed at the image plane of the imaging system (e.g., back focal plane of the TL) allowing the acquisition of in-focus images. Therefore, for reconstructing phase images of thin samples, such as the one shown in this work, there is no need to apply refocusing algorithms in the reconstruction process.

The key element of the proposed technique is the insertion of an FB^35^ between the TL and the sensor plane. Thus, at any observation plane behind the FB, there is a coherent superposition between the two split waves generated by the biprism. Remember that sinusoidal fringes are generated in the region of superposition, the rhombus-shape region with a maximum FOV, located at a distance $s_{\max}=(L/4)\tan\;\delta$ from its vertex, equal to half of the biprism’s lateral extension, ${\mathrm{FOV}}_{\max}=L/2$. Importantly, in this common-path DHM system, one only reconstructs the phase information that it is encoded within the fringes’ FOV (e.g., rhombus-shaped region). The fringes’ FOV at the sensor plane depends on the position of the FB (s), its refringence angle (δ), and its lateral extension (L). The closer the FB is to the sensor, the narrower the fringes’ FOV is. Figure 1(b) plots the experimental fringes’ FOV for an FB with δ=5 deg, L=20  mm, and refractive index n=1.5197 at a wavelength of 532 nm. As expected, the experimental data follows the trend of the theoretical prediction: the fringes’ FOV varies linearly with the FB’s position. The maximum fringes’ FOV equal 9.684 mm (error <4% compared with the theoretical prediction) is found when the relative FB position is $\Delta s_{\text{max}}=77.63\;\mathrm{mm}$ (e.g., the position between the TL and the FB is $s_{\text{max}}=s_{0}+\Delta s_{\text{max}}$). At that position ($s_{\text{max}}$), the fringes’ FOV occupies 74% of the whole sensor area, overcoming the main disadvantage of the FB-based DHM reported in Ref. 36.

It is important to highlight that the biprism generates two mutually coherent point sources that superimpose in amplitude to produce a high-contrast sinusoidal interference pattern beyond the FB. The fringes’ contrast in the sinusoidal interference pattern is directly related to the degree of temporal coherence of the real source. In other words, the use of a source with a broad spectral bandwidth produces a dramatic decay in the visibility of the fringes, resulting in a reduced fringes’ FOV. Therefore, in our FB-based DHM system, the illuminating source should have a spectral bandwidth (Δλ) no greater than 10 nm.^37^

Since our DHM system operates in the telecentric regime, the FB is illuminated by a plane wave, thus the fringes’ frequency in the FB-based DHM shown in Fig. 1(a) is independent of the biprism’s position, $p^{\prime }=1/2u_{0}$, where $u_{0}=(n-1)\tan\;\delta /\lambda$ (n,δ) are the biprism’s refracting index and refringence angle, respectively. The theoretical and experimental value of the fringes’ periods are equal to 6.08 and 7  μm, respectively. Clearly, the experimental value is close to the theoretical value. Note that for this case, the off-axis condition is satisfied because the fringes’ frequency ($u_{m}=1/pn=0.1429\;\mu m^{-1}$) is higher than $3u_{c}/2=0.0412\;\mu m^{-1}$ where $u_{c}=\mathrm{NA}/(\lambda M)=0.0275\;\mu m^{-1}$ the cut-off frequency of the imaging system (e.g., maximum spatial frequency resolvable by the system) expressed as a function of M and the NA of the system. As shown in Fig. 1(c), there is no overlay between the diffraction orders in the Fourier domain, consequently, our proposed system operates in a single-shot off-axis regime. In off-axis DHM, a better optimization of the finite space bandwidth of the sensor is achieved when the ±1 orders are placed along the diagonal of the sensor’s space bandwidth, allowing their best allocation with no overlapping. In the proposed FB-based common-path DHM system, one can ensure the best optimization of the sensor bandwidth by rotating the FB by 45 deg [Fig. 1(c)].

### 2.2 Performance Characterization

**Fig. 2.**
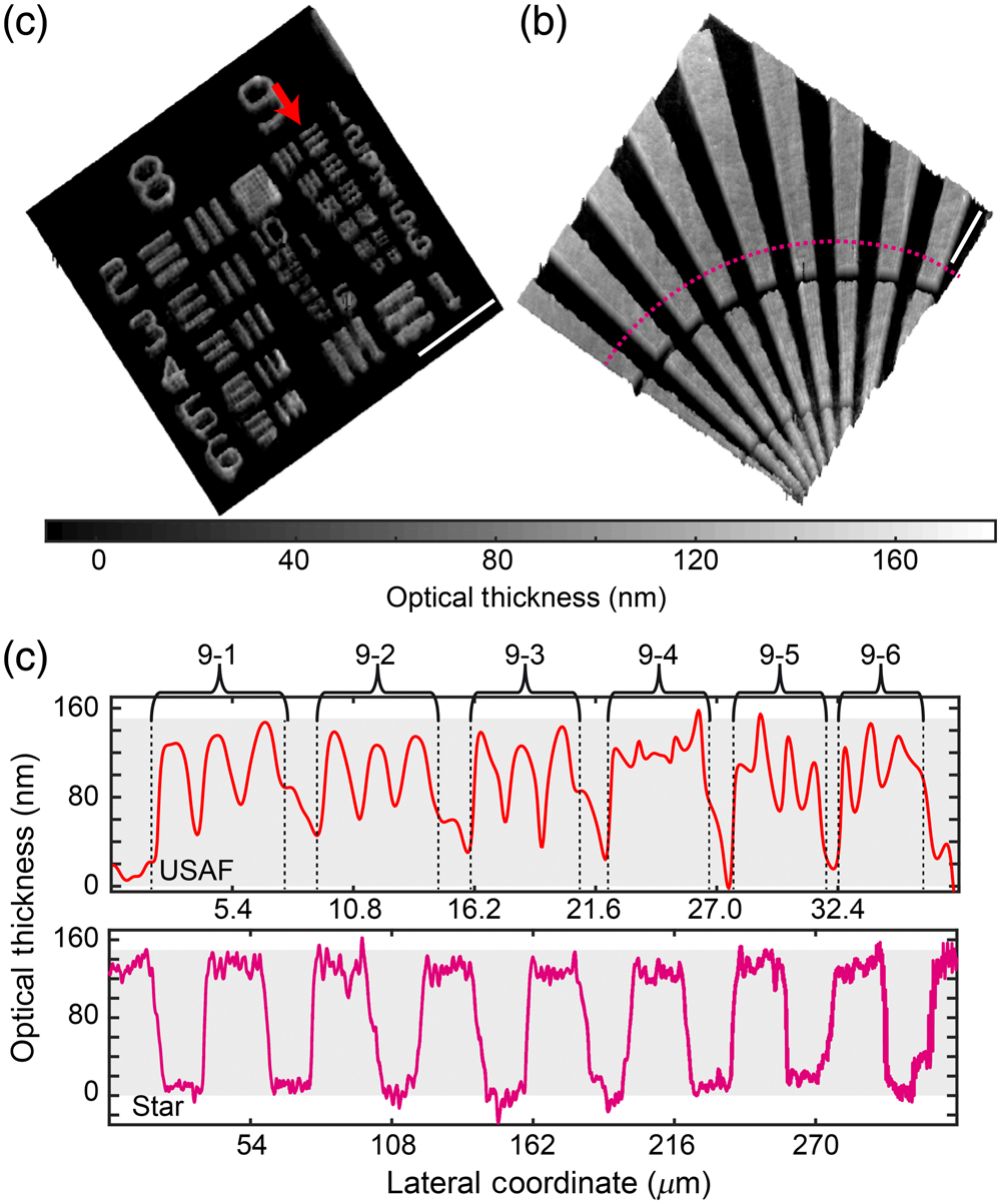
Evaluation of the FB-based common path DHM system using calibrated phase targets: (a) USAF and (b) star. 3-D reconstructed image of the targets in terms of the optical thickness. (c) Optical thickness profile of the USAF and the star target. For the USAF, the profile is taken through the horizontal lines of group 9 [direction marked by the red arrow in (a)]. For the star, the profile is taken along the radial coordinate shown in (b) (marked by the dashed pink line). The scale bar in panels (a) and (b) is 20  μm.

To evaluate the performance of the proposed FB-based common path DHM system, the FB was placed to provide the highest fringes’ FOV, thereby ensuring that the maximum sample’s FOV was reconstructed. We first used calibrated phase objects, including the USAF target and the star pattern Quality Project Management (QPM) target (Benchmark Technologies). For both targets, we arbitrarily chose the ones whose nominal heights were 150 nm. Figure 2 shows the reconstructed phase images provided by our system. Note that the measured optical thickness (t) in Fig. 2 is related to the measured phase (ϕ) from the reconstruction method via $\phi =2\pi n_{g}t/\lambda$, where $n_{g}=1.52$ is the refractive index of the glass as provided by the manufacturer. Figure 2(c) plots the optical thickness of the USAF target (top panel) and the star target (bottom panel). The USAF profile is computed along the vertical direction [marked by the red arrow in Fig. 2(a)] through the horizontal lines of group 9. For the star target, the profile was computed through the dashed pink line shown in Fig. 2(b). The measured thickness (mean±standarddeviation) for USAF and star targets, shown in Fig. 2(c), are (144±12)  nm and (131±18)  nm, respectively. Within the experimental errors, the agreement between the experimental measurement and the manufacturer’s specification [e.g., 150 nm, light gray rectangle in Fig. 2(c)] is significantly high, verifying the accuracy of the proposed FB-based common-path DHM system. We have also used the USAF image to evaluate the spatial lateral resolution limit of the proposed DHM. From Fig. 2(a), we estimate the shortest resolvable distance to be equal to 775 nm [9-3 element, marked in Fig. 2(c)], which agrees with the theoretical resolution limit for coherent imaging systems,^38^ λ/NA=818  nm. Thus, our DHM system operates at the diffraction limit.

**Fig. 3.**
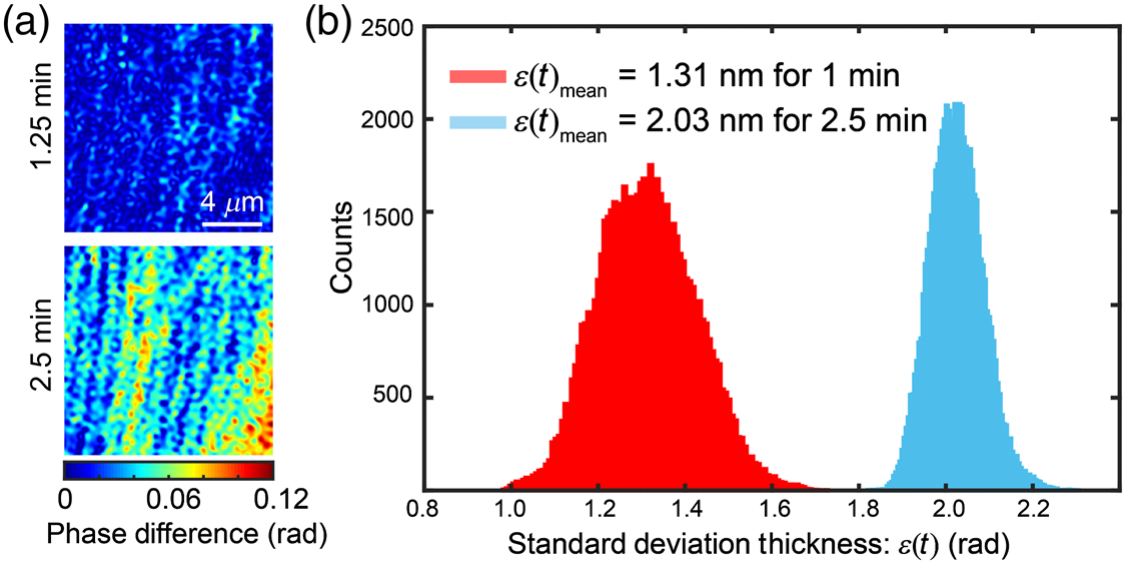
Stability results for our FB-based DHM system. (a) Phase difference map ($\phi_{t+\Delta t}-\phi_{t}$) between two frames in a time period of 1.25 and 2.5 min apart and (b) histogram of the thickness for these time periods.

The main advantage of common-path DHM systems over two-path systems is their high temporal stability. To test the temporal stability of our system, we recorded 633 holograms during 2.5 min at 4.22 fps. It is known that the stability measurement is sensitive to the experimental implementation of the system. The proposed FB-based common-path DHM system is mounted upon a floating optical table using no optical cage components. From each recorded hologram, we estimated the corresponding phase map and measured the mean value of the phase over an area of $13.96\times 13.96\;\mu m^{2}$ known-to-be-flat region. Figure 3(a) shows the phase difference map ($\phi_{t+\Delta t}-\phi_{t}$) between two frames taking at times (Δt) 1.25 and 2.5 min apart. For this FOV, the obtained phase value during the 2.5 min was 2.2765±0.0003  rad (mean±standard deviation). From the small value of the standard deviation, we conclude that our DHM system is relatively isolated from ambient fluctuations. Alternatively, other quantitative assessments of the fluctuation can be performed by evaluating the standard deviation of the phase at each pixel, ε(ϕ). Since the phase and thickness values are linearly related, one can estimate the thickness fluctuation with ε(t) being the standard deviation of the thickness from the standard deviation of the phase ε(ϕ) via $\varepsilon (t)=[\varepsilon (\phi )\lambda ]/(2\pi n_{g})$. Figure 3(b) shows the histogram of the thickness fluctuation for the two time periods. The mean value of the graph represents the thickness stability of our system, which is equal to 1.31 nm for 1 min and 2.03 nm for 2.5 min. These values confirm that the stability of the proposed common-path DHM system is within the range of a few nanometers, which enables high-resolution investigations of live specimens. Although our current implementation does not present subnanometer stability as other implementations,^14^^,^^15^ the stability can be improved using a more stable laser source and more robust implementation with cage-system assemblies. Future work will improve the system’s stability to achieve subnanometer stability.

**Fig. 4.**
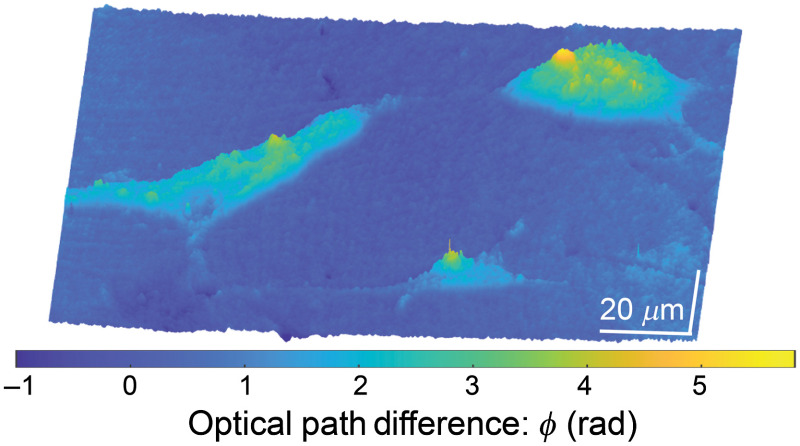
3-D pseudocolor quantitative phase images of human U87 glioblastoma cells using the proposed system. The image area is $97.21\times 140.41\;\mu m^{2}$.

The advantages of combining high speed, resolution, accuracy, and stability of the FB-based DHM system are demonstrated by imaging a monolayer of human U87 glioblastoma cells, which represent the human primary glioblastoma cell line that is commonly used in brain cancer research. The commercially available U87 glioblastoma cells (American Type Culture Collection, Rockville, Maryland, USA) are used as cancerous samples. Cells are plated on glass coverslips and inserted in a tissue culture incubator for 24 h. Then cells on covered glasses are rinsed for 5 min with phosphate buffer saline and fixed in 10% buffered formalin for 18 h at 4°C. Finally, cells are mounted on glass slides using ProLong Gold as a mounting medium. Figure 4 shows the three-dimensional (3-D) pseudocolor phase images. As a convention, we have assumed that the phase value of the glass should be equal to zero. Thereby, we have estimated the average phase value over areas free of cells (e.g., glass) and subtracted it from the total reconstructed phase value. This procedure has been applied to the unwrapped phase map using the algorithm described in Ref. 39. Clearly, Fig. 4 shows a high-contrast phase image that renders structural details in the cells distinguishing some organelles. Although this is a proof-of-concept study, this result may open the door for extending our QPI system to any biology essay.

### 2.3 Simplified Polarization-Sensitive FB-based DHM System

**Fig. 5.**
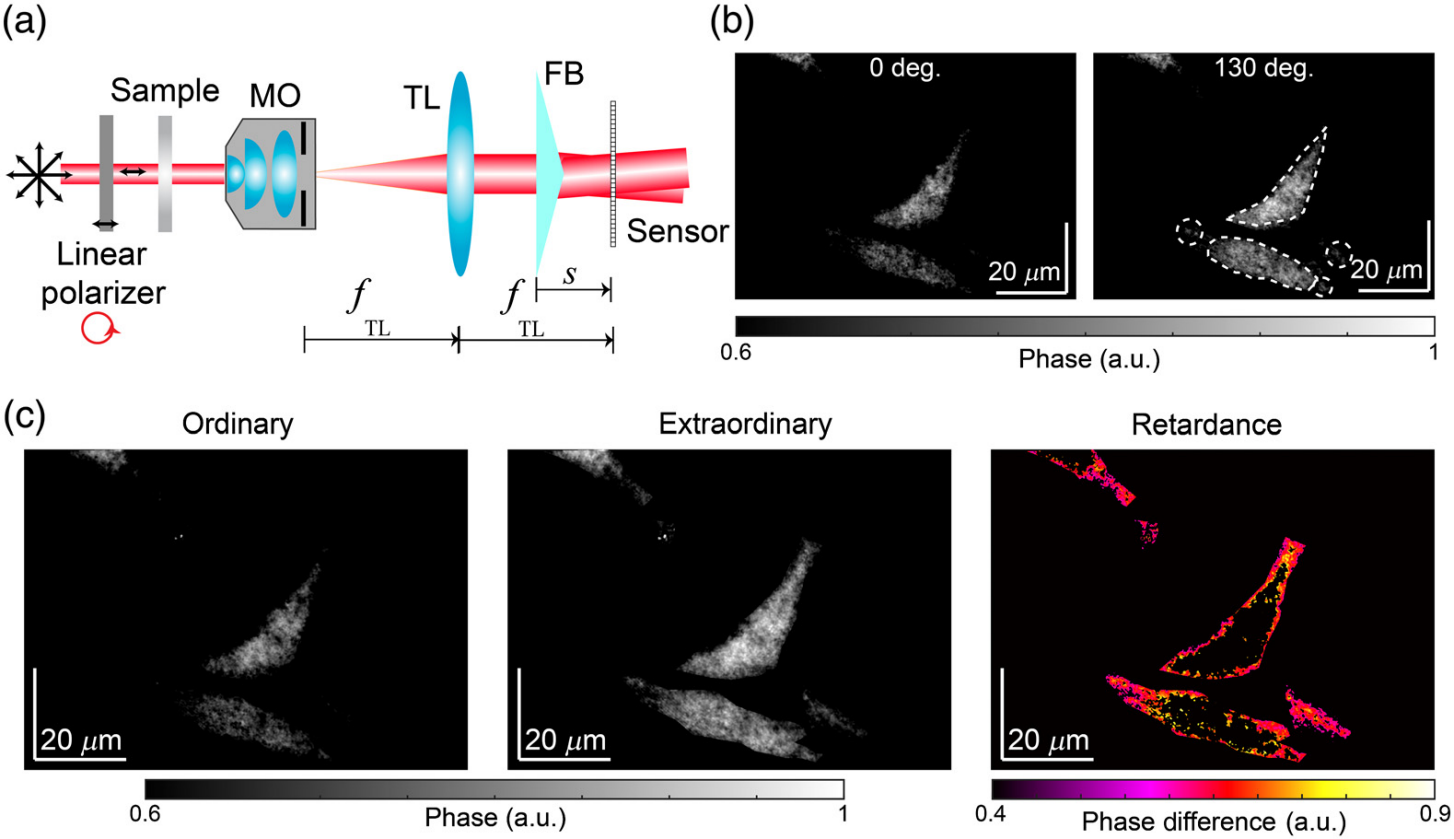
Quantitative phase images of human U87 glioblastoma cells using the proposed PS DHM system: (a) optical configuration of the simplified PS FB-based DHM system, (b) 2-D pseudocolor normalized phase maps at different polarization states (0 deg and 130 deg), and (c) ordinary and extraordinary phase maps and the retardance map.

As previously mentioned, some biological organelle and extracellular matrix components are birefringent; that is, the brightness of their image varies with the polarization state of the illuminating beam of light. To provide PS measurements (e.g., a PS-DHM system), the sample imaged by our FB-based common-path DHM system is now illuminated with linear polarized light whose plane-of-vibration is varied in a controlled way. The polarization state of the illuminating beam changes by rotating a linear polarizer inserted before the sample holder [see Fig. 5(a)]. Note that one can measure the birefringence retardance by illuminating the sample with linearly polarized light and recording the transmitted wavefront without any further analyzer polarizer.^23^ Again, we image U87 glioblastoma cells since it has been demonstrated that the glioblastoma cells present higher anisotropy than normal cells,^40^ validating the proper use of these cells for PS imaging. Figure 5(b) shows the two-dimensional (2-D) phase information from U87 glioblastoma cells illuminated with two different polarization angles, 0 deg and 130 deg. In these maps, the areas that are enclosed by the dashed lines reveal details quite differently. Some features clear for the 130-deg phase map are hardly seen for the 0-deg phase map, which confirms the PS behavior of the glioblastoma cells. To obtain the retardance map^24^ [e.g., $\Delta \phi =\phi_{e}-\phi_{o}=2\pi (n_{e}-n_{o})d/\lambda$ where $n_{e}$ and $n_{o}$ are the refractive indexes of the extraordinary and ordinary waves, and d is the thickness of the cells], we obtain the maximum and minimum values of the whole set reconstructed phase image at each pixel. Example phase maps for the extraordinary (e.g., maximum) and the ordinary (e.g., minimum) behavior are displayed in Fig 5(c). The subtraction of these unwrapped maps provides the retardance image, also displayed in Fig. 5(c). Note that on the retardance image, the contrast created is specific to the PS behavior of the sample since the cells and parts without any anisotropy are no longer visible on this image. The color scale bar in the retardance image corresponds to retardance values between 0.4 and 0.9 rad. Note that our phase sensitivity is three orders of magnitude smaller (0.0003 rad), guaranteeing that any divergence on the retardance image is due to differences of the anisotropy of the samples. Although a more rigorous research study would require the analysis of many more glioblastoma cells, our results should be regarded as a proof-of-concept study of the PS capability achieved by our system. Future work will involve the evaluation of the birefringence sensing using a benchmark birefringence target with known thickness d and birefringence or a spatial light modulator to mimic one as in Ref. 22. After this validation, further studies using several biological specimens will also be performed to evaluate the potential of this system for biological imaging.

## 3 Conclusions

We have presented a method to turn any commercial light microscope with monochromatic illumination into a PS DHM system. The proposed DHM system is based on the insertion of an FB in the image space of the microscopic imaging system. The limitations of the proposed method are the use of limited bandwidth sources (Δλ<10  nm), and, more importantly, its applicability to confluence samples such as biological tissues and high-density culture cells. Note that this limitation is always present in all self-reference DHM systems. There are different ways to ensure that there is no overlapping between the objects’ replicates generated by the biprism such as changing the axial position of biprism, replacing the biprism itself by another one with higher refringence angle, and/or using an MO lens with higher lateral magnification. In a future work, we will assess the applicability of the proposed system to image different biological samples. We will also compare our limitation with other self-reference DHM systems. Alternatively, this limitation could be address integrating our method with microfluidic devices. The FB-based common-path DHM system provides accurate quantitative PS phase images in a new, simple, compact, and cost-effective format, thanks to the low cost (a few hundred dollars) involved in implementing this simple architecture, enabling the use of this QPI technique accessible to most laboratories with standard light microscopes. We envision two possible implementations to integrate the proposed method in commercial microscopes. Because the image plane generated by the TL is inaccessible (e.g., within the microscopes’ body) in standard light microscopes, a 4f relay system is inserted to locate this image plane onto the camera sensor and/or through the eyepiece. The first possible implementation is by inserting the biprism in the image space of the second lens of the 4f relay system. Since the position of the biprism to achieve the highest FOV is independent of the objective lens used, the biprism could be mounted in a slider that the user can insert when he/she wants to provide QPI-DHM imaging. The second implementation is for microscopes that have an available side port behind the TL. In those microscopes, the proposed system could be implemented by inserting a 4f relay system attached to the side port. The main challenge of this implementation is that one must ensure that the TL lens and the first lens of the 4f relay system operate in telecentric regime. The achievement of this condition results in placing the image plane of our microscope at the back focal plane of the second lens of the 4f relay system. Note that now an auxiliary sensor should be inserted in such a way that its sensor plane is set at this new image plane. Once this relay system has been built, the biprism would be inserted between the second lens and the plane of the auxiliary system. Remember that an additional advantage of the proposed system is the reconstruction of the real 3-D information about the topography of a biological specimen. This feature cannot be accomplished with contrast-enhancing modalities of microscopy such as phase contrast, DIC, or HMC microscopy. We have also demonstrated that the utilization of an FB allows us to reconstruct accurate phase images with high spatial resolution keeping the spatial resolution limit of the imaging system from a single recorded hologram. Additionally, the temporal stability of our system is in the order of a few nanometers, being quite competitive with the state-of-art methodologies already published. Finally, with our PS FB-based common path DHM, we have observed the distinctive behavior of the cells at different polarization angles. The observed difference between the phase maps at different linear polarization states is a verification of the anisotropic properties of the glioblastoma cells. The above features make our system suitable for real-time quantitative PS phase imaging in a broad number of biological applications aimed at understanding cell growth and dynamic changes occurring during physiological processes and/or in response to pharmaceutical agents. The performance of the proposed system was verified experimentally on both calibrated phase objects and cultured cells.
